# New Issues for New Methods: Ethical and Editorial Challenges for an Experimental Philosophy

**DOI:** 10.1007/s11948-016-9838-2

**Published:** 2016-11-28

**Authors:** Andrea Polonioli

**Affiliations:** Department of Philosophy, University of Birmingham, 3 Elms Road, Edgbaston, Birmingham, B15 2TT UK

**Keywords:** Experimental philosophy, Ethics, Research integrity, Journals, Authorship, Reproducibility, Data availability

## Abstract

This paper examines a constellation of ethical and editorial issues that have arisen since philosophers started to conduct, submit and publish empirical research. These issues encompass concerns over responsible authorship, fair treatment of human subjects, ethicality of experimental procedures, availability of data, unselective reporting and publishability of research findings. This study aims to assess whether the philosophical community has as yet successfully addressed such issues. To do so, the instructions for authors, submission process and published research papers of 29 main journals in philosophy have been considered and analyzed. In light of the evidence reported here, it is argued that the philosophical community has as yet failed to properly tackle such issues. The paper also delivers some recommendations for authors, reviewers and editors in the field.

## Introduction

Philosophy is typically presented as engaged in conceptual and normative reflection, and not as an empirical discipline. Yet, whilst philosophers have been described as crucially relying on reasoning, logic, linguistic analysis, and intuitions elicited by thought experiments, a number of researchers have also argued that philosophers’ methodological toolkit should be expanded (Higgins and Dyschkant 2014). In particular, it has recently been argued for an expansion of traditional methods in philosophy by appealing to methodologies coming from the behavioral, cognitive and social sciences. More precisely, in the past 15 years, a growing number of philosophers have started to carry out, submit and publish their own empirical and experimental work (Knobe and Nichols 2008; Alexander 2012; Knobe et al. 2012; Machery and O’Neill 2014; Sytsma and Buckwalter 2016).^1^ This trend is typically referred to in the literature using the label “experimental philosophy”.^2^ In a way, by resorting to empirical methods, philosophers are not launching an entirely new tradition, but rather rescuing an older one. For instance, the image of the philosopher completely disconnected from the external world does not seem to apply to philosophers like Descartes, who put forward in his *Optics* an account of visual perception (see Sytsma and Livengood 2015).^3^

Over the past 15 years, experimental philosophy has witnessed a continuous and significant increase in attention, which is clearly demonstrated by the formation of research symposia and societies, alongside the production of special issues and collections.^4^ Yet these recent trends in philosophical research have also prompted some discussions within the philosophical community, revolving around both the philosophical significance (e.g., Cappelen 2014; Nagel and Mortensen 2016) and the scientific soundness (e.g., Cullen 2010; Seyedsayamdost 2015a, b; Strickland and Suben 2012; Huebner 2015) of experimental philosophy research.

This paper explores a set of overlooked issues emerged with the recent growth of an experimental tradition in contemporary philosophy. As a matter of stipulation, here the term “experimental philosophy” is taken to have broad extension and fuzzy boundaries, including the use of both qualitative and quantitative methods with the goal of contributing to philosophical debates.^5^ As it is argued in this paper, by importing the methods of psychology and social sciences the philosophical community has also imported a number of ethical and editorial issues that the philosophical community needs to address. These issues encompass concerns over responsible authorship, fair treatment of human subjects, ethicality of experimental procedures, unselective reporting, publishability of research findings and availability of data.

Whilst empirical disciplines have reflected on such topics for decades, and developed more or less clear guidelines on acceptable practices, the question arises as to whether the philosophical community has properly tackled, or at least reflected on, such issues. Some researchers have already considered the field of bioethics, which philosophers have traditionally been engaged with and interested in, arguing that the field lacks rigor because so many disciplines are involved, each with its own methods and standards for defining problems and establishing acceptable work (Adler and Zlotnik Shaul 2012), and because careful guidelines on research integrity are not adequately developed or followed (Resnik and Master 2011a, b). However, the questions as to whether the experimental philosophy community meets reasonable standards of rigour and whether the field of philosophy more generally has addressed the ethical and editorial issues arising from its empirical turn still seem to remain largely unaddressed.

The aim of this study is precisely to provide some evidence to assess these questions. The study considers the experimental papers published over the past 3 years in the main philosophy journals as well as philosophy journals’ instructions for authors and submission process. In light of the evidence reported, the final section also delivers a number of recommendations to authors, reviewers and editors involved in experimental philosophy work.

The paper is structured as follows. Section “The Experimental Turn in Philosophy: Emerging Issues in Philosophical Research” reviews the most pressing ethical and editorial issues that the philosophical community faces in light of the growth of an experimental philosophy tradition. Section “Operationalizing the Project” outlines a set of testable hypotheses concerning philosophers’ handling of such issues. Fourth and fifth sections discuss the “Materials and Methods” as well as the “Results” of this study. Sixth section delivers a “Discussion” of the meaning and relevance of the results, and a set of recommendations to authors and editors.

## The Experimental Turn in Philosophy: Emerging Issues in Philosophical Research

Scholarly research is *constrained* by standards of ethics and research integrity. The traditional range of research ethics, or the Responsible Conduct of Research (RCR), usually encompasses concerns over falsification, fabrication, plagiarism, and the treatment of human and animal subjects, although some researchers have also argued that there are many other ethical considerations that researchers have to deal with in their work and which are not captured by RCR (Pimple 2002; Schienke et al. 2009; Douglas 2014). Notably, however, research is also *shaped*, at least to a significant extent, by the community and journals’ editorial decisions and policies. Researchers communicate formally via peer-reviewed publications, and formal publication brings a measure of rigor and trust to this communication.^6^ A number of important editorial issues arise in empirical disciplines. Journals have to address, for instance, whether replication studies can be considered for publication or are rather considered a waste of space. Given the importance of published research outputs to a researcher’s success, it comes as no surprise that these editorial decisions end up influencing the kind of research that researchers will be carrying out.

Importantly, the range of the ethical and editorial issues relevant to the philosophical community has changed with the growth of an experimental philosophy movement. Plagiarism and conflicts of interests had typically represented the most pressing issues on research integrity in philosophical research, alongside the acknowledgement of the work of others, reasonable self-citation and distinguishing honest from careless misinterpretation (see Pritchard 1995). Interestingly, Hansson (2016) highlighted and critically discussed a number of overlooked ethical issues emerging from philosophical practice, especially in the field of moral philosophy, and Eckenwiler and Cohn (2009) have offered an examination of several ethical issues arising from research in bioethics. In a similar fashion, it seems that exploring the emerging ethical and editorial aspects connected to the growth of an experimental philosophy is also key to understanding how experimental philosophy research is both carried out and communicated. In the remainder of this section some of the issues that have arisen will be described.

### New Issues in Research Integrity

As it turns out, philosophers’ recent adoption of experimental and empirical methods is characterized by more frequent collaborative projects and co-authored papers, and this seems to raise some possible concerns. Co-authorship of papers is very common in most areas of science. To be sure, philosophical research was never classed as incompatible with collaborative work. Yet the model of the isolated philosopher traditionally accounted for a great deal of philosophical research, excluding perhaps logic and some areas of applied ethics.^7^ Now, with the rise of an experimental philosophy, this model quite clearly does not seem to be nicely applicable to this area of philosophical research. Importantly, the division of labor in experimental philosophy projects involves experiment design, data collection and performing statistical analyses. It seems safe to say that all these are rather unprecedented tasks for philosophers. Division of labor and co-authorship seem to be obvious results of these experimental trends in philosophy, and philosophers are likely to be in need of help, guidance and advice to have such tasks properly accomplished. As the importance of setting clear standards on authorship practices has been discussed with regard to the field of bioethics,^8^ it also seems that a broad discussion within the philosophical community is now in order, and that less experienced philosophers may benefit from clear instructions on criteria of authorship.

Needless to say, addressing the criteria and roles of authorship has broad and important implications to several stakeholders, such as hiring committees and funding agencies. Importantly, responsible contributorship is a delicate topic in scientific and experimental research (cf. Resnik 1997; Resnik and Shamoo 2011). In particular, there are three serious mistakes related to the assignment of credit for scientific research: assigning authorship when this is not deserved, including too many authors, and not recognize important contributions to research. So far, philosophers have done some research on co-authorship, but mostly to investigate its rationale and motivations (e.g. Bonilla 2014). Yet a deeper reflection on the meaning and implications of co-authorship in the philosophical community seems to be in order in light of the recent growth of an experimental philosophy tradition, as the latter seems to require the accomplishment of new and unprecedented tasks for philosophers and to naturally invite scientific collaborations.

### New Issues in Research Ethics

In addition, experimental studies need to comply with high ethical standards in the treatment of participants and their data, and research studies involving humans, human specimens, or human data must then follow strict protocols. Experimenters should protect the privacy and confidentiality of research subjects. Further, human subjects can participate in research only if they give their voluntary, informed consent, and during the course of the experiment the subject may stop participation for any reason and the experimenters must be prepared to stop the experiment if continuation of the experiment is likely to result in injury or distress (cf. WMA 2013). Hence, if philosophers wish to carry out, submit and publish experimental studies, they should pay careful attention to these ethical aspects of experimental work. Notably, one method of preventing unethical studies is that editors of journals state clearly in their instructions to authors that no study can be published unless the study was approved by an ethics committee and informed consent was obtained from all participants if necessary. In turn, authors should also state these points rather clearly in their manuscript.

One possible rejoinder is that philosophers should not worry too much about this ethical side of experimental research, as experimental philosophers have so far used non-invasive techniques and methodologies. After all, a great deal of experimental philosophy research consists in gathering verbal responses of adult humans to hypothetical scenarios or vignettes, also described verbally. Yet, it is first important to stress that experimental philosophers may be willing to keep expanding their methodological toolkit beyond survey-driven experimental philosophy. Notably, experimental psychologists have also encouraged this methodological expansion of experimental philosophy, stressing that the survey-based methodology is “an extremely limited research method” (Carmel 2011, 1262). Some researchers have also suggested ways to move beyond too abstract thought experiments, and for instance looked at virtual reality as more immersive environments where people can act out situations which would otherwise be difficult to construct (Wang et al. MS). These tools are more likely to produce discomfort in their users than traditional surveys.

Further, philosophers should be vigilant also when it comes to apparently non-invasive methodologies. Notably, a main interest of experimental philosophers is moral and social cognition (e.g., Kahane et al. 2012; Tobia 2015). This should come as no surprise, as moral philosophers (e.g., Foot 1967; Thomson 1985) have inspired a great deal of experimental research in experimental psychology and neuroscience (cf. Greene et al. 2001, 2008; Borg et al. 2006; Parkinson et al. 2011) and, in turn, experimental results have also been discussed for their possible relevance to and impact on philosophical theorizing (Greene 2015; Kumar forthcoming; Rini 2013; Bruni et al. 2014; Han 2014; Jeong and Han 2013; Kristjánsson 2007, 2013; Tersman 2008).

Importantly, it turns out that several stimuli used in experiments on moral judgment might actually result in participants’ distress. Consider, for instance, incest scenarios, which are a paradigmatic example of situations that evoke strong emotional reactions (e.g. Haidt 2001). Asking questions about the permissibility of incest or other potentially disturbing stimuli, perhaps especially to some particular populations or subpopulations in cross-cultural studies, might result in participants’ discomfort and distress.

Yet, the rise of an experimental philosophy also introduces concerns over possible data fabrication and falsification into the philosophical arena. These involve not only lying about the data, but also lying about how the data were generated, acquired or analyzed (Shamoo and Resnik 2009). Over the past years, a growing number of cases of data fabrication and falsification have been discovered in the natural and social sciences, and worrying reports portray a somewhat bleak picture of the ubiquity of these kinds of malpractices (Fanelli 2009). To the best of the author's knowledge, no experimental philosophy paper has so far been retracted because of data fabrication or falsification.^9^ However, there are reasons to think that data fabrication and falsification are issues that should receive philosophers’ attention. After all, the shortage of funding and resources allocated to philosophical research might have contributed to a “publish or perish” culture. In this context, it is not implausible that scholars working on experimental philosophy might be tempted to commit unethical behavior. Further, while one might expect philosophers to be more inclined to adhere to ethical behavior, this idea does not sit well with some of the available empirical evidence (Schwitzgebel 2009). But at other times unethical research and malpractice can even be subtler: less simple to put aside are actually more ordinary sorts of malpractice that can increase the likelihood of publishing false results. Multiple biases may result in inefficiency in knowledge accumulation, and scientists may take advantage of selective reporting and flexibility in analysis to make their research results more publishable (Head et al. 2015; Ioannidis et al. 2014). The philosophical community should carefully consider such possible cases of malpractice, as the rise of an experimental philosophy tradition entails the possible vulnerability of the philosophical community to such instances of misconduct.

### New issues About Editorial Policies

A number of editorial issues have recently arisen for philosophers in light of philosophy’s experimental turn. These issues concern the journals in the field of philosophy, but in turn they concern the entire philosophical community, as the editors and reviewers who run those journals come from, and represent, that very community. A first fundamental question is whether philosophy journals accept to consider empirical and experimental papers for publication. Notably, there might be different reasons for refusing to accept such papers. For instance, such papers might be deemed to lack the required philosophical insight, or the editors might believe that themselves and the journal’s reviewers lack the needed expertise to assess the scientific background. Still, if these journals decide to welcome empirical papers, another question that arises is whether direct replication studies could be accepted. For instance, it is disputed whether direct replications are more important than conceptual replications (Crandall and Sherman 2016).

Further, on top of deciding whether replication studies can be considered for publication or not, journals also need to decide which policies should be adopted to make research outputs more replicable. It is crucially important that researchers state clearly the details of the experimental procedure that has been followed. In scientific disciplines, the “Materials and Methods” section is arguably the most important aspect of a research paper because it provides the information by which the validity of a study is ultimately judged. A well-written section serves also as a set of instructions for anyone desiring to replicate the study in the future. In addition, journals can implement specific policies that encourage researchers to report all variables and conditions in a study and hence to provide methodological details regarding the paper’s reporting, making it harder to “hide” effects that did not “work” (Asendorpf et al. 2013).^10^ Further, in several corners of scientific research it has been argued that to increase reproducibility journals *should* require, as a condition for publication, that data supporting the results in the paper be accessible in an appropriate public archive or made available upon request. For instance, the Public Library of Science (PLoS) Journals, a collection of open access journals, specifically states that open access applies to both the scientific literature and the supporting data. Arguably, data sharing benefits numerous research-related activities: reproducing analyses, testing secondary hypotheses, assessing novel statistical methods, teaching, meta-analysis and, possibly, preventing error, fraud and selective reporting.

The issues that have been discussed above are “new” in philosophy and, at the same time, have clear bearing on experimental philosophy’s growth. As it turns out, the growth of an experimental philosophy movement raises a whole new set of editorial issues. Notably, issues that deal with experimental philosophy’s replicability are especially important, as some failed attempts to replicate key findings in the experimental philosophy literature have already been published (e.g., Kim and Yuan 2015; Seyedsayamdost 2015a, b). But there are further important decisions that the philosophical community needs to make. For example, if philosophers wish to publish experimental work, the field should reflect on whether Mechanical Turk or analogous crowd-sourcing services could represent efficient ways to solve the thorny problem of securing their data. In the behavioral sciences there is currently a lot of discussion going on over whether, and to what extent, these services, in which workers complete web-based tasks for small sums of money, are reliable (e.g. Crump et al. 2013; Paolacci and Chandler 2014; Hauser and Schwarz 2016). Further, besides discussing the reliability of these tools, philosophers might also want to address ethical aspects that are typically neglected: one reason why Mechanical Turk is typically deemed so “appealing” to researchers is that it can be very cheap to recruit participants, but whether underpaid work in the context of research meets criteria of fairness is open to discussion. More generally, it is important for the philosophical community and research gatekeepers to decide whether participants should receive financial incentives to take part in experiments and why financial incentives might be important (Read 2005). It should be noted that in the behavioral sciences different disciplines have different takes on the value of financial incentives (Hertwig and Ortmann 2001).

Philosophers also need to discuss the admissibility of deception in experimental papers. On the one hand, deception has traditionally been used in psychological experiments (Bortolotti and Mameli 2006), where subjects can be deceived about the purpose, design, or setting of the experiments they are participating. This tradition has been in stark contrast, for example, with the discipline of economics, where journals try to avoid publishing the results of studies that involve deception.^11^ But critics of deception came not only from the field of economics, and actually several researchers from different fields have argued that deception is not an acceptable practice (Kelman 1967; Bok 1999). Notably, things have recently started to change also in the field of psychology, as it is now common in institutional review boards for experimental psychology to limit the use of deception and require debriefing as well as other measures.^12^ But what needs to be noted is that the debate over the admissibility of deception in research is still an open one, and the philosophical community should take the issue very seriously.

## Operationalizing the Project

Once these ethical and editorial issues have been discussed, the question arises as to how it is possible to properly assess whether the philosophical community has adequately addressed them so far. One way to proceed and assess at least some of these issues would be by providing an in-depth analysis of experimental philosophy studies, for instance by conducting interviews with authors, reviewers, and editors regarding authorship criteria, ethical approval and informed consent. Clearly, there would be a lot to learn about philosophers’ handling of such issues. But whilst studies of this sort are obviously welcome, there are also evident limitations of this approach. First, this assumes that authors, reviewers and editors would be willing to accept to disclose the relevant information, where this cannot be taken for granted. Second, this approach is time-consuming and, therefore, only few studies could realistically be covered. Because of these limitations, other strategies of information extraction might be more promising.

In particular, it turns out that articles and journals should contain important information that is accessible to readers and prospective authors. Specifically, there is evidence coming from essentially three sources and that should be considered. First, it is possible to consider research outputs, i.e. published papers. These are the items available to readers, and readers will make their own assessment of the study (or of experimental philosophy more generally) based on the information contained there. Hence, important information concerning ethical approval of the experimental study, contributorship and informed consent should ideally be contained there and accessible through a targeted keyword search. Second, it is possible to consider journals’ instructions for authors or submission guidelines. Instructions for authors are arguably the main way of communication between researchers, publishers and journal editors. They serve as a readily available tool for reaching potential authors. Clearly written instructions may provide assistance throughout the whole process of manuscript preparation and, as a consequence, it is a journal’s obligation to update instructions, inform authors about editorial policies, peer review policies, code of publication ethics, manuscript preparation preferences and requirements of accompanying documents for each submission (Gasparyan et al. 2014; Horvat et al. 2015). All information concerning manuscript preparation should be readily available to authors before submitting the manuscript to the journal. Notably, however, failing to state certain policies does not entail that these policies are not applied during the editorial process. This would only indicate that those policies are not communicated to potential authors in a timely and efficient fashion.^13^ Third, information provided during a journal’s manuscript submission process can be analyzed and thereby policies that were communicated in this manner are also accessible. These three different sources of information are available to assess philosophers’ handling of ethical and editorial issues. Specifically, a set of testable hypotheses will be considered in this study.

### Experimental Papers and Replications

It is important for authors, reviewers and editors to have clear information available as to whether the journal welcomes empirical and experimental work and, if it does, whether replication studies could be considered for publication. It is hypothesized that in light of the lack of an experimental tradition in philosophy, philosophy journals fail to mention this information in their guidelines and instructions, making it difficult for authors to understand whether their contributions could be submitted or not.

### Regulation of Co-authorship

Since the philosophical community is not typically prone to co-authorship, perhaps excluding the fields of logic and applied ethics, and it is not used to division of experimental or empirical labor, it is expected that authors are not prone to provide details on respective contributions and that journals are not likely to require statements discussing authors’ contributions in the submission process or address the topic of justified authorship in their guidelines.

### Ethical Testing

Insufficient reporting of ethical issues has been discussed in different fields.^14^ Given the fact that the philosophical community is quite new to experimental research, it is expected that the situation will be more serious in philosophy journals than in non-philosophy journals. Specifically, it is hypothesized that few experimental philosophy articles will address the issues of ethical approval and informed consent, and that very few journals require statements or address the topics in their instructions for authors. Further, since organizations such as Committee on Publication Ethics (COPE; 2011) have issued recommendations and guidelines to help the editors and publishers prepare useful and informative instructions for authors, it is expected that, if journals are members of COPE, they are also more likely to address these points about ethical approval and informed consent in their instructions for authors.

### Data Fabrication and Falsification

In light of the fact that experimental philosophy is a quite recent field of research and philosophy lacks an experimental culture and tradition, it is hypothesized that philosophy journals are less prone to address the topic of data fabrication and falsification in their instructions for authors.

### Accurate Reporting

It has been recently stressed the importance of moving beyond common reporting standards to provide also methodological details that are not typically required but that are at the same time critical for accurate interpretation and evaluation of reported findings. Given the lack of experimental training and culture in philosophy, it is expected that instructions for authors will fail to mention these aspects, and that the authors of experimental philosophy papers will not be required to provide statements to explicitly confirm that all relevant information was disclosed.

### Manuscript Organization

Whilst some scientific journals clearly state the requirement to add the “Materials and Methods” section for research articles in their instructions for authors, it is expected that philosophy journals do not require or suggest any particular structure concerning articles’ sections in their instructions for authors. First, philosophers are likely to lack the sort of training in scientific writing that is typical of empirical disciplines. Second, they might believe that the philosophical implications or the justification of the study are way more important than what might appear as plain methodological minutiae.

### Data Availability

It is hypothesized that in light of philosophers’ lack of experimental culture, philosophy journals fail to either require the upload of data sets or to clarify that data should be made available upon request.

### Accepting Mechanical Turk contributions

Recruiting and testing participants is likely to constitute a significant problem for philosophers who typically lack laboratories to test participants and resources to attract them. It is thus hypothesized that a huge portion of experimental philosophy papers relies on Mechanical Turk or similar crowdsourcing platforms.

## Materials and Methods

### Study Design

A study of ethical and editorial policies of peer-reviewed philosophy journals and a cross-sectional investigation into authors’ adherence to principles of ethics and research integrity in their published research outputs have been performed.

### Sample Selection

A broad sample of peer-reviewed philosophy journals was selected. A natural way to identify the most relevant journals seemed to be by appealing to the journal impact factor (IF), which is the most common measure of a journal’s impact and quality, although its flaws are also well-known and oft-cited (Horvat et al. 2015; Brembs et al. 2013; Moustafa 2014). But IF is unavailable for most journals in philosophy and, more generally, in the humanities (Polonioli 2016). Notably, the attribution of IF depends on several factors and indexing decisions. In brief, it is not possible for a journal indexed in the Arts and Humanities Citation Index to receive an IF. The rationale behind this decision is that IFs only consider the previous 2 years, where this timeframe is taken not to be a good one for the assessment of “impact” of Arts and Humanities articles, which tend remain “relevant” for a much longer period compared to science journals.^15^ But if a journal in the humanities is indexed in the Science Citation Index and Social Science Citation Index, then it is indeed possible for that journal to apply for an IF and have it calculated. This suggests that only philosophy journals that can be listed in the Science Citation Index and Social Science Citation Index will have an IF, whereas those journals that cannot be listed there will not receive an IF.

In light of this state of affairs, two other classifications of journals were considered. One quantitative ranking is provided by the h-index metric, and it is possible to find a ranking of philosophy journals based on this last metric.^16^ But informal polls are also a popular way to rank philosophy journals, and a rather established ranking is published on the blog www.leiterreports.com.^17^ All of the journals publishing original research and included in these two rankings were considered, with these two rankings encompassing 20 journals each, and only the journal *Philosophy Compass* was excluded because it publishes only (typically invited) review articles. In light of the overlap between the two lists, the sample eventually included 29 journals, which are listed here below:

1. Nous
2. Philosophical studies
3. Philosophy and Phenomenological Research
4. Mind
5. Analysis
6. Synthese
7. Mind and language
8. Philosophers’ Imprint
9. Australasian journal of philosophy
10. Erkenntnis
11. Review of Philosophy and Psychology
12. Ergo
13. Philosophical Review
14. Philosophical Quarterly
15. Canadian Journal of Philosophy
16. Philosophical Psychology
17. Ethics
18. Journal of Philosophy
19. Phenomenology and the Cognitive Sciences
20. Journal of Consciousness Studies
21. Philosophical Perspectives
22. Ratio
23. Journal of Philosophical Logic
24. Pacific Philosophical Quarterly
25. American Philosophical Quarterly
26. Studies in Philosophy and Education
27. European Journal of Political Theory
28. Proceedings of the Aristotelian Society
29. European Journal of Philosophy

A group of psychology journals that could be used as benchmark for a comparison was also identified. Different rankings are popular in psychology and philosophy. Notably, in psychology, unlike philosophy, impact factors are typically calculated and often taken into account when choosing whether one particular outlet is better than another. In establishing the sample, those psychology journals that had been considered for the recent investigation of the reproducibility of psychological studies (Open Science Collaboration 2015) were firstly included, as these are considered to be the most important and impactful venues. The sample was then doubled by including three randomly selected journals publishing original research in experimental psychology^18^ from the first tier of the IF ranking^19^. Still, to preserve some of the heterogeneity not only in terms of impact (broadly construed) and rejection rate, but also in terms of publishing and business model that was found in the list of philosophy journals, the only open access journal publishing psychological work was also included in the sample:^20^

1. Journal of Experimental Psychology: Language, Memory and Cognition (IF 2.862)
2. Journal of Experimental Psychology: General (IF 5.929)
3. Psychological Science (IF 4.940)
4. Cognition (IF 3.479)
5. Journal of Personality and Social Psychology (IF 5.031)
6. Frontiers in Psychology (IF 2.560 and Open Access)

#### Part 1

The 4589 research articles published in the past 3 years in the 29 philosophy journals in our sample were accessed and analyzed, isolating experimental/empirical papers using a broad search strategy. Review articles, book reviews, errata, editorials, and corrigenda were not considered when looking at the research articles published in the past 3 years. The PDF version of the articles was accessed and the coder searched for the keywords ‘experiment,’ ‘empirical,’ ‘subject(s),’ ‘participant(s),’ ‘sample,’ ‘test,’ and ‘statistic(al).’ Also the words ‘interview’, ‘poll’, ‘case study’ and ‘survey’ were considered, to detect qualitative research articles: as stated in the previous sections, an assumption of this paper is that experimental philosophy includes qualitative research.^21^ On cases where the keyword-based search strategy was deemed to be less effective to discriminate between empirical research and literature reviews, a quick read of the paper was also applied. 122 out of those 4589 articles were identified as experimental articles. Also the 64 experimental papers published in the last issue by the 6 psychology journals selected as sample (*Frontiers in Psychology* publishes articles continuously, without “journal issues”, and 11 just published articles were taken as sample) were considered.

At this point, the 122 experimental philosophy articles were considered to determine whether the authors had addressed the issues of ethical approval and informed consent by searching for the terms ‘ethic(al)’, ‘board’, ‘approved’, ‘Helsinki’, ‘committee’, ‘informed’, ‘consent’. The coder then searched for studies using the terms ‘contribut(ed),’ ‘authorship,’ ‘collected,’ or ‘analysed/zed” to look for mentions of authorship and authors’ contributions. The same was done with the 64 experimental psychology articles to obtain a benchmark against which to compare the results obtained in the case of philosophy papers. For the experimental philosophy articles the number of citations received so far was checked by referring to *Google Scholar* as database: whilst this database is not necessarily the most accurate, as it is rather (arguably, too) liberal, other databases such as *PhilPapers*, *Scopus, Web of Knowledge* or *PubMed* are inadequate for this analysis as they either miss many citations outside the field of philosophy (*Philpapers*) or within the field of philosophy (*Scopus, Web of Knowledge* or *PubMed*). The average number of citations per article can be used to shed further light on the impact and relevance of the sub-field of experimental philosophy. Finally, a search for the terms “Mechanical turk”, “M-turk” and “turk” was also conducted to determine whether the studies constituting the sample had relied on such a tool. Since similar crowd-sourcing services that are not offered by Amazon cannot be detected through this keywords based search, the estimate to be obtained is conservative.

#### Part 2

Besides analyzing the research outputs published in philosophy journals and comparing them against psychology ones, further evidence was also considered by reviewing the instructions for authors of these journals, as well as the information provided to authors during the submission process. Whilst previous studies (e.g. Asai and Shingu 1999; Bosch et al. 2012; Strech et al. 2014; Horvat et al. 2015) had typically only focused on instructions for authors, it turns out that quite often the relevant information is required during the later (submission) stage. Notably, three of the 29 journals did not allow the coder to access information concerning the submission process, as they either have submission windows and the access was not allowed (*Philosophy and Phenomenological Research* and *Nous*) or required submission fees (*Philosophers’ Imprint*). The following 15 questions were also considered when looking at the evidence:

1. Is the journal a member of COPE?
2. Does the journal inform authors of whether experimental papers can be submitted?
3. Does the journal inform authors of whether replication studies could be submitted?
4. Do the instructions for authors address the topic of justified authorship?
5. Do the instructions for authors address the topic of ethical approval?
6. Do the instructions for authors address the topic of informed consent?
7. Do the instructions for authors address the topic of falsification or fabrication?
8. Do the instructions for authors address the topic of data availability?
9. Do the instructions for authors provide indications on article’s suggested sections and structure?
10. Do the instructions for authors provide indications on fair and accurate reporting?
11. Are the authors asked to provide statements of authorship and contributorship upon submission?
12. Are the authors asked to confirm ethical approval upon submission?
13. Are the authors asked to confirm that informed consent was obtained upon submission?
14. Are the authors asked to confirm that their reporting of the experimental study is accurate upon submission?
15. Are authors asked to upload data sets upon submission?

Notably, to answer question (1) the COPE webpage (http://publicationethics.org/members) was also considered, as not all journals that are members of COPE do list that information on their website.

### Statistical Analyses

Chi square tests of independence and Fisher’s exact tests, where appropriate, were performed to explore the association between subject type (i.e. Philosophy and Psychology) and relevant variables. They were also used to explore whether there was a relationship between whether or not a philosophy journal belonged to COPE and whether the journal addressed responsible authorship, ethical approval, and informed consent in the instructions for authors.

Point-biserial correlations were used to assess the relationship between number of citations and presence of author statement, ethical statement and reference to participants’ informed consent, and between number of authors and author statement, ethical statement and reference to participants’ informed consent. All analyses were performed using SPSS for Mac v.20.

### Coding Reliability

To ensure that idiosyncratic biases and coding errors did not affect the assessment of articles and journals, a second coder also coded articles from a random 23% of the experimental philosophy papers and 23% of the psychology papers considered by the primary coder (the author), as well as journals from a random 24% of the philosophy journals and 33% of psychology journals considered by the primary coder.

## Results

Of the 122 experimental philosophy articles, 16 (13%) included ethical statements and 8 (7%) mentioned informed consent by participants. Also, 4 (3%) included authorship statements. In addition, 48 of them (39%) referred to use of Mechanical Turk. Finally, the average citations for these experimental papers was 8.65, and 32 (26%) of the 122 experimental articles had more than 10 citations.

Further, of the 29 philosophy journals examined, 18 (62%) were members of COPE. Five of them (17%) seem to clarify whether they welcome empirical and experimental work but none of them addressed whether replication studies could be submitted. Interestingly, even the journal *Erkenntnis*, which lists a non-trivial number of traditions favorably considered by the journal, avoids referring to experimental philosophy and experimental studies:

Erkenntnis […] concentrates on […]:

Epistemology

Philosophy of science, foundations and methodology of science in general and of natural and human sciences such as physics, biology, psychology, economics, social sciences in particular

Philosophy of mathematics

Logic, philosophy of logic, and all kinds of philosophical logics

Philosophy of language

Ontology, metaphysics, theory of modality

Philosophical psychology, philosophy of mind, neurophilosophy

Practical philosophy, i.e. ethics, philosophy of action, philosophy of law, etc.^22^

Moreover, the journal *Synthese*, whilst explicitly mentioning that formal approaches are accepted, fails to mention whether experimental work is welcome:

Synthese is a philosophy journal focusing on contemporary issues in epistemology, philosophy of science, and related fields. More specifically, we divide our areas of interest into four groups: (1) epistemology, methodology, and philosophy of science, all broadly understood. (2) The foundations of logic and mathematics, where ‘logic’, ‘mathematics’, and ‘foundations’ are all broadly understood. (3) Formal methods in philosophy, including methods connecting philosophy to other academic fields. (4) Issues in ethics and the history and sociology of logic, mathematics, and science that contribute to the contemporary studies Synthese focuses on, as described in (1)–(3) above.^23^

Further, 10 (34%) of these journals addressed in their guidelines the topic of responsible authorship, 7 (24%) referred in their guidelines to ethical approval and informed consent. Also, 8 (28%) of the journals addressed in their guidelines the issue of data availability and 7 (24%) addressed the topic of data fabrication or falsification. Yet, none of the journals gave indications on fair reporting of procedures and results or on manuscript’s suggested sections. Notably, whilst philosophy journals do sometimes offer guidance on sections’ formatting, they fail to suggest a particular structure for articles or to demand that they include particular sections such as a “Materials and Methods” one in case of empirical work. The journal *Analysis*, for instance, only writes that “in case of longer papers, it can be helpful to divide the piece into numbered sections, and the sections may also be given headings”.^24^ Only 3 (12%)^25^ of the journals addressed the topic of ethical approval during submission stage, and none of the journals referred to responsible authorship, fair reporting or participants’ informed consent during the submission stage. Finally, none of the philosophy journals required upload of data sets.

The results reveal a statistically significant association between COPE membership and whether journals’ guidelines for authors addressed the topic of responsible authorship [Fisher’s exact test, *p* = .044; Odds Ratio (OR) = 10.00] and included reference to ethical approval (Fisher’s exact test, *p* = .026; OR_adj_ 26 = 15.00) and informed consent (Fisher’s exact test, *p* = .026; OR_adj_ = 15.00). On the other hand, there was no statistically significant correlation between number of citations and whether authors addressed the topic of responsible authorship (*r*_pb_ = −.072, 95% BCa CI [−.163, .013], *p* = .434, two-tailed) and included reference to ethical approval (*r*_pb_ = .084, 95% BCa CI [−.095, .303], *p* = .357, two-tailed), although a statistically significant correlation was found between number of citations and reference to participants’ informed consent in the paper (*r*_pb_ = .304, 95% BCa CI [−.035, .596], *p* = .001, two-tailed). The correlation between the number of authors and whether authors addressed the topic of responsible authorship was statistically significant (*r*_pb_ = .295, 95% BCa CI [−.010, .502], *p* = .001, two-tailed). There were no statistically significant correlations between number of authors and whether authors included reference to ethical approval (*r*_pb_ = .132, 95% BCa CI [−.067, .318], *p* = .147, two-tailed), and whether articles included reference to participant’s consent (*r*_pb_ = .151, 95% BCa CI [−.056, .341], *p* = .097, two-tailed).

Chi square tests of independence were performed to explore the association between subject type (i.e. philosophy and psychology) and relevant variables as outlined below. The results of the Chi square tests revealed that the proportion of articles containing ethics statement [*X* ^2^ (1, *N* = 186) = 16.45, *p* < .001; φ = .297], reference to informed consent [*X* ^2^ (1, *N* = 186) = 32.62, *p* < .001; φ = .419] and authorship statement [*X* ^2^ (1, *N* = 186) = 40.84, *p* < .001; φ = .469], to those that did not, differed significantly between philosophy and psychology articles (Table 1).

**Table 1:** Percentages of authorship and ethical statements, as well as reference to informed consent in philosophy and psychology papers

	Philosophy papers (*n* = 122) (%)	Psychology papers (*n* = 64) (%)
Ethics statement	13	39
Consent	7	41
Authorship statement	3	39

Moving to the analysis of journals (Table 2), there were statistically significant differences between philosophy and psychology journals considering whether or not the journal referred to acceptability of empirical/experimental work (Fisher’s exact test, *p* < .001; OR_adj_ = 57.91) and replications (Fisher’s exact test, *p* = .003; OR_adj_ = 59.00), as well as whether they referred to ethical approval (Fisher’s exact test, *p* = .001; OR = 39.00), informed consent (Fisher’s exact test, *p* = .001; OR_adj_ = 39.00), availability of data (Fisher’s exact test, *p* = .019; OR = 13.13), and indications on manuscript’s sections (Fisher’s exact test, *p* = .003; OR_adj_ = 59.00) in their instructions for authors. Further, there were statistically significant differences between the field of the journal and whether or not the journal referred to ethical approval (Fisher’s exact test, *p* = .002; OR = 38.33), informed consent (Fisher’s exact test, *p* < .001; OR_adj_ = 194.33), responsible authorship (Fisher’s exact test, *p* = .030; OR_adj_ 29.44), and fair reporting (Fisher’s exact test, *p* = .030; OR_adj_ = 29.44) during the submission process. On the other hand, the percentage of journals that are members of COPE compared to those that are not was not significantly different between philosophy and psychology journals (Fisher’s exact test, *p* = .367; OR = 0.31). Also the proportion of journals addressing responsible authorship (Fisher’s exact test, *p* = 1; OR = 0.95), data fabrication and falsification (Fisher’s exact test, *p* = .322; OR = 3.14), and fair reporting (Fisher’s exact test, *p* = .171; OR_adj_ = 16.09) in their guidelines did not differ significantly between philosophy and psychology journals. There was also no statistically significant difference in the percentage of journals requesting authors to upload their data sets during the submission process between philosophy and psychology (Fisher’s exact test, *p* = .188; OR_adj_ = 14.45; Table 2).

**Table 2:** List of aspects and issues by which journals were analyzed and detailed information about differences in the philosophy and psychology groups

	Philosophy journals (*n* = 29)^a^ (%)	Psychology journals (*n* = 6) (%)
COPE membership	62	33
Responsible authorship discussed in guidelines	34	33
Need of ethics approval discussed in guidelines	24	100
Data availability requirements discussed in guidelines	28	83
Indications on manuscript structure in the guidelines	0	50
Acceptability of experimental work stated	17	100
Acceptability of replication studies stated	0	50
Guidelines on informed consent	24	100
Guidelines on data fabrication	24	50
Guidelines on fair reporting	0	17
Information on ethics approval requested upon submission	12	83
Confirmation of responsible authorship requested upon submission	0	33
Confirmation of fair reporting requested upon submission	0	33
Upload of raw data requested	0	17
Information on informed consent requested upon submission	0	83

^a^For the last five categories, data was available only for 26 Philosophy journals

Finally, considering the assessment provided by the second coder, the reliability of coding was extremely high. Specifically, in the case of the random 23% of experimental philosophy papers scrutinized by a second coder as well, inter-rater reliability was perfect for the assessment of information concerning use of Mechanical Turk, authorship statements and ethics statements (Cohen’s *k* = 1), and substantive in the case of information regarding informed consent (Cohen’s *k* = 0.78). Further, in the case of the random 23% of psychology articles examined also by a second coder, inter-rater reliability was perfect (Cohen’s *k* = 1) for the assessment of information concerning authorship statements, ethics statements and participants’ informed consent. With regard to the assessment of the instructions for authors and submission processes of the random 24% of the philosophy journals and 33% of the psychology journals that were also analyzed by a second coder, the agreement between raters was also extremely high. In particular, in the case of philosophy journals, reliability was perfect (Cohen’s *k* = 1) for the assessment of journals’ COPE membership as well as of information in the guidelines concerning need of ethical approval and informed consent, availability of data, responsible authorship, acceptability of empirical/experimental work, and data fabrication/falsification. Cohen’s *k* could not be computed due to lack of variability in codes for at least one of the raters for the assessment of instructions on manuscript’s structure, acceptability of replications and fair reporting, as well as for the analysis of requests of raw data upload and ethical approval, confirmation of fair reporting, responsible assignment of authorship and participants’ informed consent upon submission. Still, in all of these cases there was full agreement between coders, except in the case of the assessment of requests of ethical approval during the submission process, where there was disagreement on just 1 out of 7 journals examined. In addition, in the case of psychology journals reliability was perfect (Cohen’s *k* = 1) for the assessment of information on COPE membership as well as of instructions on data fabrication/falsification, manuscripts’ structure, responsible authorship, acceptability of replication studies, and fair reporting. Cohen’s *k* could not be computed due to lack of variability in codes for at least one of the raters for the assessment of guidelines on responsible authorship and participants’ informed consent, ethical approval, data availability, acceptability of experimental work and fair reporting, as well as for the analysis of requests of ethical approval, participants’ informed consent and upload of raw data during the submission process. Still, in all of these cases there was full agreement between coders, except in the case of the assessment of instructions on responsible authorship, where there was disagreement on 1 journal. Finally, the count of citations provided by the two coders was not analyzed statistically because their coding did not take place at the same time, and discrepancies were therefore expected.

## Discussion

This study provides insight on philosophers’ adherence to current recommendations in scientific publishing and compared philosophers’ practices to those found in the field of psychology. A first important finding of this study is that whilst experimental papers are still not published very frequently in philosophy, they are rather influential. The average number of citations (8.65) is indeed impressive if compared to the impact factor of the few philosophy journals that have such number available, and even to the impact factor of psychology journals. For instance, the journal *Philosophical Studies* has an IF of 1.256, and the psychology journal *Cognition* has an IF of 3.634.^27^ This finding in itself further strengthens the rationale for my focus on the philosophical community’s preparedness to handle experimental papers and its adherence to principles of ethics, research integrity and good scholarship.

This work provides evidence for insufficient reporting of ethical and research integrity issues in experimental philosophy papers. Quite worryingly, this was the case also for the “most influential” papers: articles with a higher number of citations were not more likely to have authorship and ethical statements and to refer to participants’ informed consent than those with fewer citations. Notably, whilst experimental philosophers have declared that they wish to import the methods and tools of psychologists, it seems that they have not as yet followed in psychologists’ footsteps when it comes to adherence to research integrity and ethics issues.^28^ This result is rather important: insufficient reporting of ethical issues within experimental philosophy research can negatively affect how trustworthy the public judges the philosophy research community to be. Public trust in the research community requires evidence that this specific community has qualities such as competence and good will that merit that trust. Insufficient reporting of ethical issues may not only give the impression to the public but also to the research community itself that the ethical quality of research is judged far less important than its scientific validity.

The fact that research outputs do not provide relevant information concerning ethics and research integrity is consistent with a scenario in which philosophy journals have updated and well-informed guidelines and authors fail to follow the advice and instructions. But it is also consistent with a different scenario in which journals fail to provide authors with relevant information and instructions. Notably, this study also sheds light on which of these two scenarios best describes the current situation of philosophy journals. On the one hand, it turns out that most scientific journals have made important efforts to provide authors with accurate guidance on ethics, research integrity and good scholarship. But philosophy journals have not properly updated their instructions and requirements yet. These, again, are worrying outcomes. Failure to update journals’ instructions could be read as signifying that these journals and, in turn, the philosophical community, do not regard these as serious issues. A more encouraging result, however, is that, as predicted, COPE members have so far implemented more accurate and detailed guidelines and requests for authors. It should be noted that, in part, poor control of submissions’ adherence to standards of ethics and research integrity could be explained also by the fact that many philosophy journals (***n*** **=** 8)^29^ require that papers be submitted via email; arguably, this submission procedure makes it more difficult for editors to have control over the article’s metadata and seems more conducive to losses of attached materials and information.

Arguably, however, the study has also some limitations. First, there are some experimental philosophy articles that have been published in journals such as *Episteme*, where the latter outlet was not part of the sample. Second, some journals that are classed here as ‘philosophical’ are actually outlets intended to attract genuinely interdisciplinary research, such as the case of the *Review of Philosophy and Psychology*. Third, more refined analysis could be obtained by also ‘rating’ the quality of instructions provided to authors. In other words, the applied coding strategy (score “0” for answer “no” and score “1” for answer “yes”) implies that certain requests that were elaborated in detail in some instructions for authors and just briefly mentioned in others would be equally scored. Similarly, articles’ statements could also vary significantly in their quality and accuracy. Fourth, the keyword-based search for information is clearly a non-perfectly reliable way to obtain information. At the same time, accuracy/efficiency trade-offs seem to strongly justify its use. To maintain acceptable levels of accuracy, however, the coders reviewed the texts themselves, compensating with some more careful reading when the keyword-based search was ineffective, instead of simply resorting to unsupervised automated methods for exploiting the information available in the papers (e.g., Boyack et al. 2011). Fifth, information stated in the supplementary materials was not analyzed. Sixth, to paint a complete picture on whether some particular policies are enforced by psychology journals it would be best to have some further information. For instance, authors are supposed in the case of three of the six psychology journals to fill out, sign and send to the editor a “Certification of compliance with APA ethical principles”, but we have no evidence showing that this practice is actually enforced.

Still, this exploration of conformity to principles of ethics, research integrity and good scholarship in the philosophical community does license a verdict that is at the same time quite clear and rather worrying. It seems that the philosophical community has so far failed to properly address the new constellation of ethical and editorial issues that philosophy’s experimental turn has raised. In particular, *qua* authors philosophers have not shown adequate sensitivity to issues of research integrity and ethics. Moreover, *qua* editors philosophers have failed to provide adequate guidance and address concerns over responsible authorship, fair treatment of human (and-non human) subjects, ethicality of experimental procedures, availability of data, unselective reporting and publishability of research outputs. This is in turn a problem for journals’ referees, who are research gatekeepers but cannot find in the journals’ guidelines clear advice on fundamental aspects of research integrity and quality. In light of this it seems that philosophy might not have the best practices in place when it comes to review papers that use empirical methods.

Notably, this result might look particularly striking if we consider how much attention philosophers have devoted to some other sets of issues potentially affecting the transmission of knowledge. More precisely, journals have tried to find ways to fights biases in peer-review. There have long been complaints that peer review can be unfair. For instance, in the common ‘single-blind’ system, there are concerns about bias, knowing or unknowing, on grounds of sex, race, nationality, or field of study. Philosophy journals have typically looked at double-blind review systems as ways to tackle this problem. In some cases, even triple-blind peer review. For instance, the journal *Mind* states on its website that “the review at *MIND* is ‘triple-anonymous’—the identity of authors is not revealed to editors or referees unless and until a paper is accepted for publication”. Importantly, the philosophical community has devoted a lot of its attention to issues of fairness in review, but somewhat neglected the other crucial issues that have been explored in this paper, which might nevertheless be interfering with the quality of knowledge communication and that have long been addressed by other empirically-oriented research communities.

The situation can be changed, obviously. It is recommended that journal guidelines and instructions in philosophy journals be accurately and promptly updated. Editors should clarify their policies, perhaps publishing editorials to discuss their in-house handling of such issues. Yet, until this is done, authors might also want to consider submitting experimental philosophy papers to psychology journals (as a non-trivial number of researchers have actually already done), which have proven to be better equipped to deal with empirical work. Notably, some psychology journals such as Frontiers in Psychology (via the section Theoretical and Philosophical Psychology) and Theoretical and Philosophical Psychology explicitly welcome philosophical contributions.^30^ Should philosophers wish to continue and submit experimental philosophy papers to philosophy journals, some recommendations seem to be in order. Experimental philosophers could try and send pre-submission inquiries to philosophy journals editors or their editorial office to verify whether experimental papers and replications could be submitted. Importantly, prospective authors should also start and provide all the relevant information in their manuscripts and try to adhere to standards of good research in the first place. Further, there are also platforms that might help experimental philosophers’ job. For instance, *Psychdisclosure* (http://psychdisclosure.org) is an open-science initiative that provides a platform for authors of recently published articles to disclose methodological design specification details that are not typically required under common reporting standards but that are at the same time critical for accurate interpretation and evaluation of reported findings. All in all, it seems that carefully reflecting on these aspects will be an important initial step towards the development of a more informed and considerate empirically-minded research community.

## Footnotes

1 Note, however, that the use of formal methods, on top of experimental ones, has also been vigorously defended in the philosophical arena (cf. Hartmann et al. 2013).

2 Notably, however, the use of the term "experimental" is controversial, as most of the research associated with it actually involves surveys only. Still, following Prinz (2008), it has been popular to draw a distinction between “empirical philosophy”, namely just empirically-informed philosophical research, and “experimental philosophy”, where philosophers actually try to directly provide some empirical evidence to test their hypotheses.

3 I wish to thank the editor of this journal for suggesting this point.

4 It might be worth mentioning that philosopher De Cruz recently made the case for the importance of launching a journal in experimental philosophy: http://philosopherscocoon.typepad.com/blog/2016/04/journal-of-experimental-philosophy-expressions-of-interest.html.

5 Here simulation studies are not classed as part of “experimental philosophy”. The definition and alleged epistemic privilege of experiments over simulations are important topics of research that will not be addressed here (Guala 2002; Morrison 2009; Frigg and Reiss 2009; El Skaf and Imbert 2013; Parke 2014). Suffice it to say for the purpose of this paper that computer simulations do not seem to raise the same ethical and editorial challenges that other research methodologies do.

6 It is quite commonly claimed that research communities in the humanities place greater emphasis on vehicles of communication that are not journal articles, such as monographs or book chapters (e.g., Eve 2014). Arguably, however, at least in the case of philosophy journal articles constitute a fundamental type of research output.

7 I thank an anonymous reviewer for highlighting this point.

8 Let us note, however, that it is not entirely clear how to conceive of the relationship between bioethics and philosophy.

9 Cf. http://retractionwatch.com/?s=philosophy. Some caution is in order when trying to interpret data on numbers of retractions (Fanelli 2013). It is still worth reminding, however, that retractions due to duplicate publication or plagiarism have already occurred even in the field of philosophy (for a brief discussion, see Hansson 2016, pp. 2–3).

10 For initiatives in this spirit see, e.g., http://psychdisclosure.org.

11 For instance, the *American Journal of Agricultural Economics* states in its website that “if the protocol involves deception of human subjects, document the extent and nature of deception involved and the research justification for inclusion of the deception. The handling editor may return manuscripts in which the deception is deemed unwarranted or excessive given the research justification, even if the protocol was approved by all appropriate Institutional Review Boards.” In the field of economics, this ban on deception is not justified based on ethical principles.

12 I wish to thank an anonymous referee for suggesting that I mention this trend.

13 Notably, however, how much freedom editors have when it comes to updating their journals’ instructions might depend on the publisher and vary from case to case.

14 Recent studies in different fields have shown that instructions to authors in journals often fail to require authors to state that the study was approved by an ethics committee and informed consent obtained from participants; articles also often omit mentioning these points (cf Weil et al. 2002; Strech et al. 2014).

15 Others stress that “in arts and humanities, the typical level of journal-to-journal citations is so low that citation-based journal metrics are generally not used in these fields” (Morris et al. 2013, p. 151).

16
https://scholar.google.nl/citations?view_op=top_venues&hl=nl&vq=hum_philosophy. Notably, different metrics are also available. For instance, journals indexed in Scopus by Elsevier can also be ranked according to Scimago metrics (cf González-Pereira et al. 2010).

17 (http://leiterreports.typepad.com/blog/2015/09/the-top-20-general-philosophy-journals-2015.html). Notably, journals officially refer to their placement on this ranking. The journal Analysis writes that “*Analysis* has been ranked 10th in the Leiter Reports' Top Twenty 'General' Philosophy Journals” to advertise its quality: http://analysis.oxfordjournals.org.

18 For instance, *Annual Review of Psychology* was then excluded because it publishes reviews only.

19 See, for instance goo.gl/NGqAw0.

20 In particular, *Ergo* and *Philosophers’ Imprint* were, for example, platinum and gold open access journals respectively. This sets them aside from the bulk of other journals. *Frontiers in Psychology* in the sample of psychology journals is a gold open access journal.

21 Regarding this inclusion of qualitative research, see also Andow (forthcoming).

22 See http://www.springer.com/philosophy/journal/10670.

23
http://www.springer.com/philosophy/epistemology+and+philosophy+of+science/journal/11229.

24
http://www.oxfordjournals.org/our_journals/analys/for_authors/.

25 The percentage is calculated based on the 26 journals for which information concerning the submission process was available.

26 OR_adj_ differs from OR in that it includes an adjustment method to overcome the so-called problem of empty cell (see, e.g., Agresti 1990, 2002). More precisely, when a cell has zero frequency in a table, it is computationally impossible to obtain Odds Ratio as an estimate of effect size. Here I follow Grissom and Kim (2012) in adding a constant .5 to each cell as a solution to the problem.

27 Whilst the calculation of a journal’s IF seems rather straightforward to calculate, it has been argued that the data used in the calculation of the impact factor are neither transparent nor publically available (Rossner et al. 2007).

28 Importantly, here I am not arguing that the psychology community has already successfully tackled these issues concerning ethics, research integrity and good scholarship. After all, the several retraction scandals (e.g., Brown et al. 2013; Ruys and Stapel 2008) and outcomes of the replicability project (Open Science initiative 2015) keep reminding us of some serious outstanding problems. But at least these topics are typically addressed and carefully discussed.

29 More precisely, I am referring to: *Journal of Consciousness Studies*, *Analysis*, *Journal of Philosophy*, *Mind and Language*, *Pacific Philosophical Quarterly*, *Philosophical Quarterly*, *Proceedings of the Aristotelian Society* and *Philosophical Perspectives*.

30 I wish to thank an anonymous referee for suggesting this point.
